# Pre-Exposure to Stress-Inducing Agents Increase the Anticancer Efficacy of Focused Ultrasound against Aggressive Prostate Cancer Cells

**DOI:** 10.3390/antiox11020341

**Published:** 2022-02-09

**Authors:** Hakm Y. Murad, Partha K. Chandra, Charles A. Kelly, Namrata Khurana, Heng Yu, Emma P. Bortz, Shirley N. Hong, Debasis Mondal, Damir B. Khismatullin

**Affiliations:** 1 Department of Biomedical Engineering, Tulane University, New Orleans, LA 70112, USA; hmurad@tulane.edu (H.Y.M.); ckelly8@tulane.edu (C.A.K.); hyu3@tulane.edu (H.Y.); ebortz@tulane.edu (E.P.B.); shong2@tulane.edu (S.N.H.); 2 Tulane Institute for Integrative Engineering for Health and Medicine, Tulane University, New Orleans, LA 70112, USA; 3 Department of Pharmacology, Tulane University, New Orleans, LA 70112, USA; pchandr1@tulane.edu (P.K.C.); NKhurana@mdanderson.org (N.K.); 4 Department of Microbiology, DeBusk College of Osteopathic Medicine, Lincoln Memorial University, 9737 Cogdill Road, Knoxville, TN 37932, USA; 5 Tulane Cancer Center, Tulane University, New Orleans, LA 70112, USA

**Keywords:** prostate cancer, aggressive phenotype, oxidative stress, ER-stress, focused ultrasound, CDDO-me, nelfinavir, combined mechanochemical disruption

## Abstract

Despite the initial success in treatment of localized prostate cancer (PCa) using surgery, radiation or hormonal therapy, recurrence of aggressive tumors dictates morbidity and mortality. Focused ultrasound (FUS) is being tested as a targeted, noninvasive approach to eliminate the localized PCa foci, and strategies to enhance the anticancer potential of FUS have a high translational value. Since aggressive cancer cells utilize oxidative stress (Ox-stress) and endoplasmic reticulum stress (ER-stress) pathways for their survival and recurrence, we hypothesized that pre-treatment with drugs that disrupt stress-signaling pathways in tumor cells may increase FUS efficacy. Using four different PCa cell lines, i.e., LNCaP, C4-2B, 22Rv1 and DU145, we tested the *in vitro* effects of FUS, alone and in combination with two clinically tested drugs that increase Ox-stress (i.e., CDDO-me) or ER-stress (i.e., nelfinavir). As compared to standalone FUS, significant (*p* < 0.05) suppressions in both survival and recurrence of PCa cells were observed following pre-sensitization with low-dose CDDO-me (100 nM) and/or nelfinavir (2 µM). In drug pre-sensitized cells, significant anticancer effects were evident at a FUS intensity of as low as 0.7 kW/cm^2^. This combined mechanochemical disruption (MCD) approach decreased cell proliferation, migration and clonogenic ability and increased apoptosis/necrosis and reactive oxygen species (ROS) production. Furthermore, although activated in cells that survived standalone FUS, pre-sensitization with CDDO-me and/or nelfinavir suppressed both total and activated (phosphorylated) NF-κB and Akt protein levels. Thus, a combined MCD therapy may be a safe and effective approach towards the targeted elimination of aggressive PCa cells.

## 1. Introduction

Prostate cancer (PCa) is the second leading cause of cancer-associated morbidity and mortality in elderly men in the United States [1]. The majority of these patients are also at a higher risk of complications from the currently available treatment approaches such as surgery and radiation therapy [2]. Due to the side-effects associated with these invasive approaches, many patients choose to defer treatment of less aggressive tumors. In order to suppress the risk of tumor progression, these patients often undergo androgen deprivation therapy (ADT) which also presents with significant long-term complications [3,4]. Furthermore, the castration-resistant PCa cells (CRPC) acquire an aggressive and metastatic phenotype by activating androgen-independent signaling pathways [5]. Indeed, PCa progression to metastatic CRPC (mCRPC) results in a mortality of ~40% of patients within 10 years [6,7]. Therefore, development of better treatment strategies is critical for the long-term survival of patients with early-stage and localized PCa. 

Focused ultrasound (FUS) has shown significant promise in its ability to eliminate tumors, enhance the efficacy of cancer immunotherapy and suppress the recurrence of an aggressive tumor phenotype [8]. FUS causes mechanical disruption of tumor cells and their microenvironment due to intense molecular vibrations that alter protein configuration and break intermolecular bonds [9,10,11,12]. As a result of the FUS-induced mechanical disruption and localized increase in temperature, tumor cells experience stress that results in their apoptosis and necrosis. The long-term benefits of tumor elimination using FUS is also being tested in numerous clinical laboratories [13]. Transrectal FUS is emerging as a safe alternative to radiotherapy and prostatectomy in the treatment of early-stage (T1-T2a) PCa [14]. Although transrectal delivery of high-intensity FUS (referred to as HIFU) is an approved technique in PCa patients, the side effects associated with these high intensities (up to 20 kW/cm^2^) have dampened the initial promise of FUS as a targeted and noninvasive tumor-elimination therapy [15,16]. At acoustic intensities ≥0.9 kW/cm^2^, FUS induces anti-proliferative and apoptotic effects on tumor cells [17], but it also elicits side effects on normal cells [15], and effects on vascular endothelial cells adjacent to the tumor have been documented [16]. However, at lower intensities (0.1–1.0 W/cm^2^), FUS is not cytotoxic and has, in fact, been shown to stimulate cell proliferation, as demonstrated in studies with bone marrow stem cells and peripheral neuronal cells [18,19]. In addition, recent evidence shows that standalone HIFU may promote tumor recurrence and progression in aggressive liver cancer [20] and cannot prevent rapid tumor recurrence in ~50% of patients with intermediate-risk (T2b) or high-risk (T2c-T4), localized PCa [21,22]. Therefore, it is envisioned that the utility of moderate FUS doses (0.1–0.9 kW/cm^2^) may have profound clinical application towards a targeted elimination of localized PCa, and novel strategies to enhance the anticancer efficacy of FUS are clearly warranted. 

Our previous *in vitro* and *in vivo* studies indicated that the curative potential of FUS is increased when cancer cells are co-exposed to chemical agents that interfere with cellular response to mechanical disruption, e.g., ethanol or sorafenib [17,23,24]. Our current observations show that a similar mechanochemical disruption (MCD) to sensitize aggressive PCa cells to FUS can be achieved by co-exposure to drugs that increase oxidative stress (Ox-stress) and dysregulate redox signaling, i.e., CDDO-me [25,26], and/or drugs that increase endoplasmic reticulum stress (ER-stress) and are known to target autophagy, i.e., nelfinavir [27,28]. Aggressive PCa cells exploit multiple stress-signaling pathways to overcome the effects of cytotoxic therapy and adapt to hostile tumor microenvironments, and a continuous balance in Ox-stress and ER-stress pathways is crucial to tumor survival. Therefore, we proposed that chemical agents that disrupt these stress-signaling pathways may increase the killing efficacy of mechanical disruption by FUS. Towards this goal, we employed a drug-repurposing approach by choosing two pharmaceutical agents that are known to increase Ox-stress and ER-stress in cancer cells and are in several anticancer clinical trials, CDDO-me and nelfinavir [29,30].

The first compound tested towards tumor cell sensitization prior to their FUS exposure was CDDO-me, a C-28 methyl ester of 2-cyano-3,12-dioxoolean-1,9-dien-28-oic acid, which dysregulates redox signaling and rapidly increases reactive oxygen species (ROS) production. CDDO-me is currently in late-stage clinical trials for chronic kidney disease (CKD), and numerous past studies, by us [31] and others [25,26], have documented its potent anticancer effects at nanomolar (nM) concentrations. The second pharmaceutical agent we chose to use towards pre-sensitization of PCa cells prior to FUS exposure was nelfinavir (Viracept^TM^), a clinically approved, anti-HIV drug that is also in several anticancer clinical trials. Numerous studies, by us [32] and others [27,28], have shown that nelfinavir increases multiple markers of ER-stress and autophagy in aggressive cancer cells. Both of these clinically tested agents are also known to suppress two crucial survival mechanisms in tumor cells, i.e., the NF-κB and Akt pathways [33,34]. Therefore, our primary objective was to investigate whether pre-treatment with low doses of CDDO-me and/or nelfinavir can sensitize PCa cells and increase the tumor-eliminating ability of FUS. 

## 2. Materials and Methods

### 2.1. Cell Culture

Four PCa cell lines, purchased from American Type Culture Collection (ATCC, Rockville, MD, USA), were used in this study. These were LNCaP (CRL-1740), C4-2B (CRL-3315), 22Rv1 (CRL-2505) and DU145 (HTB-81) cells [32,35,36,37]. LNCaP cells are AR-positive and androgen-dependent and serve as an *in vitro* model of early-stage PCa that are responsive to ADT [32]. C4-2B cells are a subline of LNCaP, developed by continuous growth under castrate conditions and are used as a model of CRPC cells. These cells show ligand-independent AR signaling and constitutive nuclear AR levels and can metastasize to bones in *in vivo* mouse models [35]. 22Rv1 cells are also a CRPC line and show constitutive, androgen-independent AR activation via high-level expression of the AR splice variant, AR-V7 [36]. The DU145 line represents highly metastatic PCa cells and was originally isolated from a patient with brain metastases. These cells do not express AR and grow well under castrate conditions *in vivo* [37]. All the above four cell lines were cultured in high-glucose DMEM (Thermo Fisher Scientific, Waltham, MA, USA) supplemented with 10% fetal bovine serum (Thermo Fisher Scientific) and 1% penicillin/streptomycin (Thermo Fisher Scientific) at 37 °C and under 5% CO_2_. They were at passage number 5–8 when used in experiments.

### 2.2. Focused Ultrasound

A 1.1 MHz, single-element, concave acoustic transducer (H102, Sonic Concepts, Bothell, WA, USA) was used in all experiments. A 33220A function generator (Agilent Technology, Santa Clara, CA, USA) produced an input sinusoidal signal that passed through a fixed-gain (50 dB) ENL 2100L power amplifier (Electronics & Innovation, Rochester, NY, USA) and then entered the transducer. The transducer had a stainless-steel housing with active diameter of 64 mm, which was coupled to a cone containing degassed water heated to 37 °C. The FUS signal strength was monitored using a 2 Giga-samples/s InfiniVision DSO-X-2014A oscilloscope (Agilent Technology, Santa Clara, CA, USA). Temperature near a tumor sample was measured during FUS targeting by a mini-hypodermic Copper Constantan type T 200 μm thick bare-wired thermocouple (Omega Engineering, Stamford, CT, USA) connected to a temperature meter (SDL200, Extech Instruments, Waltham, MA, USA). FUS was operated in a continuous mode. 

The acoustic intensities used in our current study were less than half the intensity of current, clinically approved HIFU, which is ~1.5 kW/cm^2^ [38]. Experiments on the effects on cell proliferation were conducted with FUS at acoustic output power of 8.7 W (level H4) for 30 s. This power level corresponds to the spatial peak temporal average intensity (I_SPTA_) of 0.70 kW/cm^2^. In the scratch-wound assays, to ensure that cells could migrate post treatment, FUS was operated at an acoustic power of 2.7 W (level H2; I_SPTA_ = 0.24 kW/cm^2^). 

### 2.3. Experimental Procedure

Eight treatment groups were measured in this study: (a) untreated (control); (b) FUS alone; (c) CDDO-me (50–400 nM); (d) nelfinavir (2 µM); (e) nelfinavir + CDDO-me; (f) CDDO-me + FUS; (g) nelfinavir + FUS; and (h) nelfinavir + CDDO-me + FUS. Briefly, designated concentrations of CDDO-me and/or nelfinavir were added to cells (2.7 × 10^6^) in fresh growth medium. Following 24 h incubation, the medium was collected, and cells were trypsinized and centrifuged (2000 rpm for 2 min), and the cell pellet was placed in a thin-wall 0.2 mL centrifuge tube (Bio-Rad, Hercules, CA, USA) containing 100 µL of the previously collected medium. Resuspended cells were then exposed to FUS at acoustic output power of either 0.24 kW/cm^2^ for the scratch-wound assays (H2) or 0.70 kW/cm^2^ for all the other assay conditions (H4). 

### 2.4. Cell Proliferation Assay

Cell proliferation assays were carried out according to our previous publication [32]. Approximately 1.0 × 10^5^ untreated or treated cells (as mentioned above) were cultured in 0.1 mL of growth medium in a 96-well flat-bottom plate (Fischer Scientific, Pittsburgh, PA, USA). Their proliferation rate was measured at both 24 h and 72 h post treatment by using the WST-8 Cell Proliferation kit (Cayman Chemical, Ann Arbor, MI, USA). Briefly, medium-containing plates were incubated with 10 µL of WST-8 reagent, and absorbance at a wavelength of 540 nm was measured using a microplate reader (ELx808, BioTek Instruments, Winooski, VT, USA). 

### 2.5. Flow Cytometry

Flow cytometry assays were carried out according to our previous publication [17,23]. Cells exposed to drugs and/or FUS were grown in 35 × 10 mm^2^ culture dishes. Viable, early apoptotic and late apoptotic/necrotic cell populations were measured at different time points by a flow cytometric Annexin V-FITC assay (Thermo Fisher Scientific). Briefly, cells in each treatment group were first washed with ice-cold PBS and ×1 binding buffer. They were then incubated with 5 µL annexin V in a 195 µL cell suspension at room temperature for 15 min and washed twice with the binding buffer. A 10 µL amount of propidium iodide (PI, 20 µg/mL) was added to the cell suspension immediately prior to flow cytometry. A total of 100,000 events, excluding aggregates and particulates, in the forward and side-scatter gates were collected using the Attune Acoustic Focusing Cytometer (Applied Biosystems, Grand Island, NY, USA). Early apoptotic cells were identified as PI-negative and annexin-V-positive, whereas late apoptotic/necrotic cells were positive for both PI and annexin V. 

### 2.6. Hanging Drop Culture

Hanging drop cultures were carried out according to our previous publication [23]. The untreated and treated cells (3.0 × 10^4^ per mL) were seeded into wells of Perfecta3D™ Hanging Drop Plates (3DBiomatrix, Ann Arbor, MI, USA) and imaged over the course of three days. Images of multicellular spheroid cultures were captured, and spheroid formation ability was blindly measured by five independent scorers using the following metrics: 0 = no spheroids, 1 = loosely packed spheroids and 2 = tightly packed spheroids. 

### 2.7. Scratch-Wound Assay

Scratch-wound assays were carried out according to our previous publication [31]. Cells were seeded in 6-well plates (1 × 10^6^ per well) and grown until they formed a confluent monolayer, and monolayers were scratched (wound) using a 200 µL pipette tip. Wells were then washed with PBS, and images of the wound area at 0 time point were captured using a CCD camera on a Leica microscope (Buffalo Grove, IL, USA). Growth medium was added back to each culture, and treatments were initiated. Images of the scratched monolayer were taken at different time points post treatment, and scratch width was measured as a distance between 4–5 random points within the wound edges by using the ImageJ software (NIH). 

### 2.8. Colony-Forming Unit Assay

Colony-forming unit assays were carried out according to our previous publication [31]. Cells were seeded in 60 mm petri dishes (500 cells/dish) 24 h post exposure to drugs and/or FUS. They were cultured in growth medium supplemented with 2% FBS for 14 days, after which the medium was removed, and each dish was washed with PBS. Colonies were then fixed with formalin for 30 min and stained with 0.2% methylene blue for another 30 min. Excess methylene blue was washed off with deionized water. Dishes were imaged, and cell colonies were counted using ImageJ. 

### 2.9. ROS Measurement

Oxidative stress measurements were carried out according to our previous publication [31]. Production of reactive oxygen species (ROS) in the untreated and treated cells was measured by using a chloromethyl (CM) derivative of H2DCFDA (Thermo Fisher Scientific). Briefly, cells were incubated with 100 μM CM-H2DCFDA for 2 h before treatment and for up to 72 h post treatment. Immediately before ROS measurement, cells were first washed with ice-cold PBS and resuspended in the growth medium. The ROS-associated CM-H2DCFDA fluorescence (excitation at 495 nm and emission at 520 nm) was measured by flow cytometry. 

### 2.10. Immunoblot Assay

Primary mouse antibodies against human NF-κB p65 (clone A-12) and phosphorylated (Ser536) NF-κB p65 (clone 27) were obtained from Santa Cruz Biotechnology (Santa Cruz, CA, USA). Primary rabbit antibodies against human pan-Akt (clone 44-609G), phosphorylated (Ser473) Akt1 (clone 14-6) and those against glyceraldehyde 3-phospahate dehydrogenase (GAPDH) were obtained from Thermo Fisher Scientific. Western immunoblot assays were carried out according to our previous publication, with minor modifications [31]. Total protein was extracted from both untreated and treated cells by using radio-immunoprecipitation assay (RIPA) lysis buffer and quantified using the bicinchoninic acid (BCA) protein assay reagent (Thermo Fisher Scientific). Equal amounts of protein (50 µg/lane) were electrophoresed on 8–12% NuPAGE gels (Thermo Fisher Scientific) and transferred to Immobilon-P membrane from EMD Milipore (Billerica, MA, USA). After blocking nonspecific binding using 5% casein in 1× TBS-T buffer (Tris buffer saline with 0.1% Tween-20), membranes were incubated with primary antibodies (1:5000 dilution) at 4 °C overnight. Membranes were then washed with TBS-T buffer and incubated with horseradish peroxidase (HRP)-conjugated goat anti-mouse or goat anti-rabbit secondary antibody (LI-COR Biosciences, Lincoln, NE, USA) at 1:10,000 dilution in bovine serum albumin (BSA)/TBS-T solution. Incubation was carried out in a dark room at room temperature for 2 h. The molecular weight of proteins was estimated using the PageRuler Prestained Protein Ladder (Thermo Fisher Scientific). Protein band images were acquired by Odyssey Infrared Imager (LI-COR Biosciences) and analyzed by Image Studio software (LI-COR Biosciences).

### 2.11. Statistical Analysis

Results were evaluated with a paired or unpaired two-tailed *t*-test, one-way ANOVA with Tukey post-test or two-way ANOVA with Bonferroni post-test by using GraphPad Prism (GraphPad Software, La Jolla, CA, USA). An unpaired *t*-test with Welch’s correction was used for datasets with different sample sizes and/or unequal variances. The normal distribution of data was confirmed by the Shapiro–Wilk test. Statistically significant differences were set to *p* < 0.05 between experimental groups. The statistical data are represented as mean ± standard error of the mean (SEM), and the number of independent tests (n) is listed in each figure legend. 

## 3. Results

### 3.1. Pre-Sensitization of LNCaP Cells with CDDO-Me Increases the Cytotoxic Efficacy of FUS

**Figure 1:**
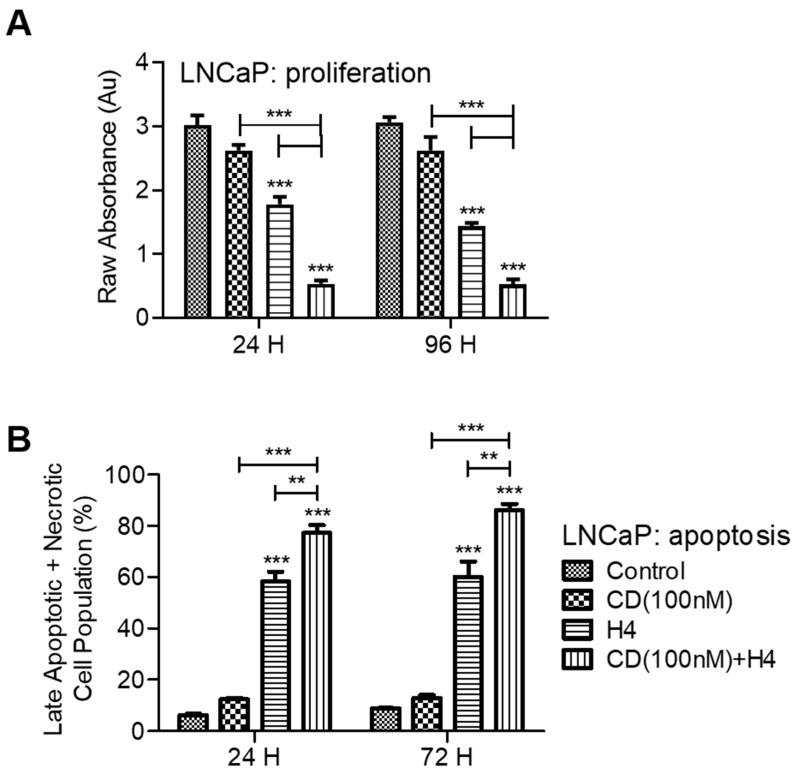
Combination effect of FUS and CDDO-me on proliferation and apoptosis in LNCaP cells. In (**A**), cell proliferation (WST-8 assay) at 24 h and 96 h post treatment with CDDO-me (100 nM) + FUS (H4) is shown. In (**B**), the percentage of late apoptotic and necrotic cells at 24 h and 72 h post treatment with CDDO-me and FUS is shown. Bar graphs show ± SEM of 3–4 independent experiments. Significant differences from untreated controls or individual treatment groups are represented as *p*-values; ** *p* < 0.01 and *** *p* < 0.001. Pre-sensitization of LNCaP cells with low-dose CDDO-me increased the cytotoxicity of FUS.

The LNCaP cells express AR and require androgen for their growth. Using these cells, we first tested the effects of pre-sensitization with low-dose CDDO-me (100 nM) on FUS- mediated cell viability and proliferation (Figure 1A). Results obtained at both 24 h and 96 h post exposure to FUS showed that combined treatment with CDDO-me and FUS enabled higher suppressive effect on cell proliferation. Exposure to CDDO-me showed <20% decrease in cell viability, and FUS alone caused ~50% decrease in cell growth. However, in CDDO-me (CD) pre-treated LNCaP cells, exposure to FUS (H4) enabled a more than 80% decrease in cell viability. Enhancement of the cytotoxic efficacy of FUS in CDDO-me pre-sensitized LNCaP cells was then documented by flow cytometry (Figure 1B). As compared to FUS or CDDO-me alone, co-exposure significantly increased the percentage of late apoptotic and necrotic cells, as measured at both 24 h and 72 h post treatment. These initial observations in the androgen-dependent LNCaP cells indicated that subtoxic doses of CDDO-me can be used to enhance the anticancer efficacy of moderate-intensity FUS. Further studies were carried out using three CRPC cell lines, C4-2B, 22Rv1 and DU145, to understand the molecular mechanisms. 

### 3.2. Pre-Sensitization with CDDO-Me Increases FUS-Induced Oxidative Stress and Enhances Cytotoxicity in C4-2B Cells 

**Figure 2:**
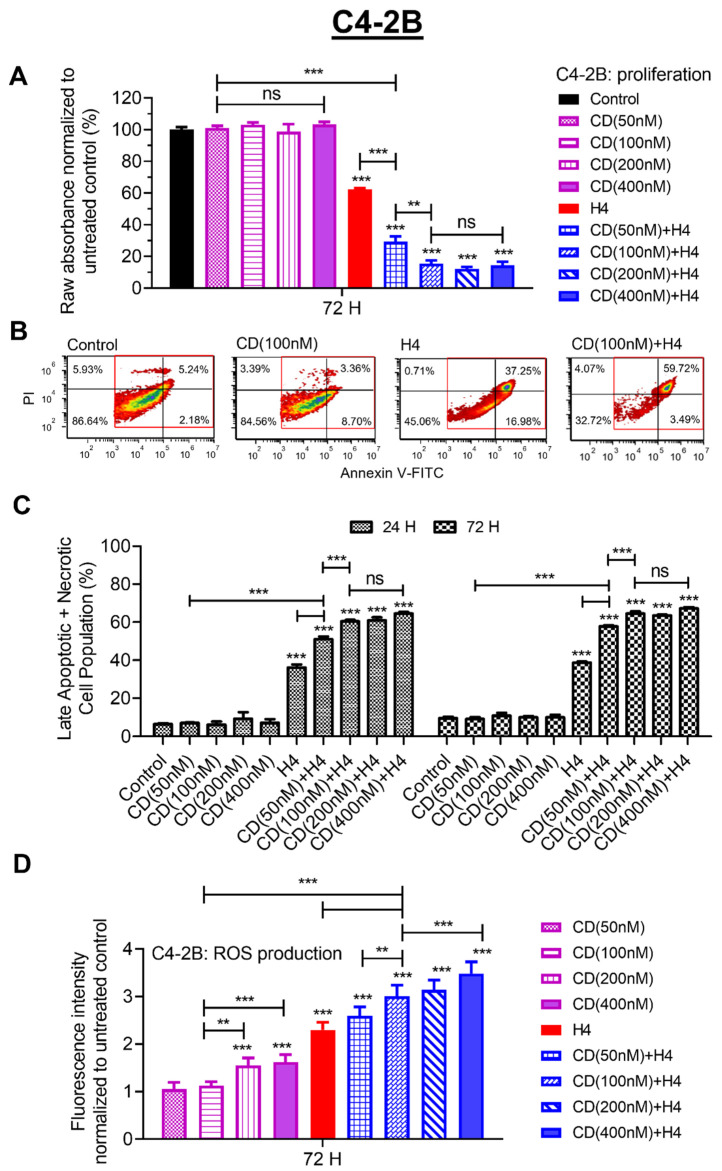
Effect of CDDO-me on FUS-induced suppression of cell viability and increase in oxidative stress in C4-2B cells. Panel (**A**) shows the concentration-dependent effect of CDDO-me (50–400 nM), alone and following exposure to FUS (H4), on C4-2B cytotoxicity at 72 h post treatment. Bar graphs show the absorbance (OD), and significant changes are shown as *p*-values (** *p* < 0.01; and *** *p* < 0.001). ns represents ‘not significant’. (**B**) flow cytometry analysis of C4-2B cells exposed to CDDO-me (100 nM) or FUS (H4), alone and in combination. The percentage of early apoptotic/necrotic cells (PI+/FITC+) is shown in the upper right quadrant. (**C**) the late apoptotic/necrotic C4-2B cells, as a percentage of total cell population. Effect of increasing concentrations of CDDO-me (50–400 nM), alone and in combination with FUS (H4), at both 24 h (solid) and 72 h (hatched) is shown in the bar graphs (*** *p* < 0.001). (**D**) ROS production by C4-2B cells measured using the fluorescent dye H2DCFDA. Bar graphs depict the effect of CDDO-me (50–400 nM), alone and following exposure to FUS (H4), at 72 h post treatment. Bar graphs show the normalized fluorescence, and significant changes are shown as *p*-values (** *p* < 0.01 and *** *p* < 0.001).

The C4-2B cells are an aggressive subline of LNCaP and possess a CRPC phenotype. In this cell line, we first investigated the dose-dependent effect of CDDO-me (50–400 nM) on cell viability in the absence or presence of FUS. The 72 h absorbance data clearly showed that low-dose CDDO-me (CD) was not toxic to the C4-2B cells, but its pre-treatment was able to significantly increase the killing ability of FUS (Figure 2A). Standalone FUS showed a <50% decrease in cell viability, which increased to as much as 85–95% when cells were pre-sensitized with CDDO-me. Even the lower doses of CDDO-me (50 and 100 nM) were able to sensitize C4-2B cells to the cytotoxic effects of FUS. Flow cytometry analysis using propidium iodide (PI) and annexin V (FITC) was carried out to measure the effects on early and late apoptosis. At early stages of apoptosis, the cells bound annexin V and excluded PI, and, at late stages of apoptosis, they stained brightly with both annexin V and PI. Results with combination treatment clearly showed an increase in early-stage apoptosis, as evident from 37.25% death with FUS alone to 59.72% death with the FUS + CD combination (Figure 2B). The bar graphs in Figure 2C show the flow cytometry results obtained with increasing doses of CDDO-me (50–400 nM) either in the absence or presence of FUS (H4) at both 24 h and 72 h post exposure.

FUS-induced molecular changes in cells are known to increase oxidative stress and ROS production [11,14,38]. Furthermore, at micromolar concentrations, the cytotoxic effects of CDDO-me have also been linked to increased ROS generation [25,26,39]. Therefore, we measured ROS production in C4-2B cells at 72 h post treatment with CDDO-me (50–400 nM) in the absence or presence of FUS by using the ROS-sensitive dye, H2DCFDA (Figure 2D). Fluorescence intensities obtained in CDDO-me- and/or FUS-exposed cells were normalized to the untreated controls. A dose-dependent increase in ROS production was seen in CDDO-me-treated C4-2B cells at 72 h, with the higher concentrations (200 nM and 400 nM) showing a ~20% increase. Exposure to FUS at level H4 (0.70 kW/cm^2^) alone increased ROS production by almost 2-fold, and a significantly higher ROS level was evident in cells that were pre-treated with CDDO-me. In fact, the FUS-induced ROS production was significantly higher than that observed in cells that were exposed to even the low concentrations of CDDO-me, and a more than a 3-fold increase of FUS-induced oxidative stress was documented in cells that were pre-treated with the higher doses of CDDO-me. These findings indicated that the enhanced killing ability of the FUS + CDDO-me combination may be associated with higher ROS production by these aggressive PCa cells. Hence, further studies were carried out to investigate whether this treatment combination could suppress the aggressive phenotype of C4-2B cells. 

### 3.3. Pre-Treatment with CDDO-Me Enhances FUS-Mediated Suppression of Aggressive Phenotype and Decreases NF-κB Induction in the Surviving C4-2B Cells 

**Figure 3:**
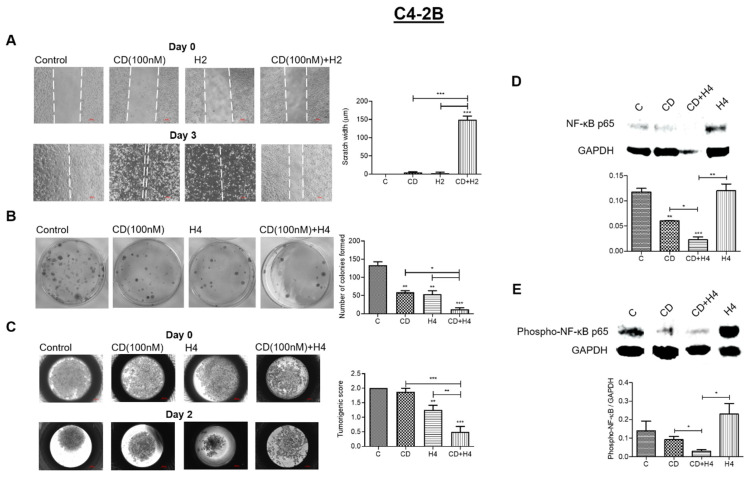
Effect of combination treatment on migration, colony formation, spheroid formation and both total and activated NF-κB levels in C4-2B cells. Scratch-wound assays were carried out to measure the effect of low-dose FUS (H2), alone and in combination with CDDO-me (100 nM), at day 3 post treatment (**A**). Representative images of day 0 (top) and day 3 (bottom) wound width are shown on the left. Bar graph data on the right show wound width at day 3 (*n* = 4), indicating a significant (*p* < 0.001) suppression in migratory properties of C4-2B cells following combined treatment. In (**B**), CFU assays were carried out in C4-2B cells exposed to CDDO-me (100 nM) or FUS (H4), alone and in combination. Representative images of colonies are shown on the left, and bar graph data (*n* = 3) are shown on the right. Combined treatment resulted in a significant suppression of C4-2B colony-forming ability. (**C**) effect of combination treatment on spheroid formation by C4-2B cells at day 2 of the spheroid culture. Representative images of tightly or loosely packed spheroids are shown on the left, and bar graph data (*n* = 8) are shown on the right. In (**D**,**E**), effects of combined treatment on both total and activated (phosphorylated) NF-κB p65 protein levels are shown. Representative images of NF-κB protein levels are shown in the top panel, and the loading control (GAPDH) is in the bottom panel. Bar graphs in both (**D**,**E**) are the data of three independent experiments (*n* = 3). Significant changes from untreated controls or individual treatment groups are represented as *p*-values (* *p* < 0.05; ** *p* < 0.01; and *** *p* < 0.001).

Aggressive cancer cells possess an increased ability to evade cytotoxic therapy, migrate and form 3-dimensional (3-D) tumor colonies within distant niches [40,41]. We wanted to investigate whether combined exposure to FUS and CDDO-me enables a higher suppression in these aggressive properties in C4-2B cells as compared to either of these treatments alone. Effects on cell migration were measured by using scratch-wound assay (Figure 3A), effects on colonizing ability were measured by CFU assay (Figure 3B) and effects on 3-D spheroid formation were measured by using hanging drop culture assay (Figure 3C). As evident in Figure 3A (left panel), in the untreated cultures (control), C4-2B cell migration at day 3 enabled complete closure of the wound. However, exposure to either CDDO-me (100 nM) or FUS at level H2 (0.24 kW/cm^2^) slightly decreased cell migration, as evident from the increases in wound width. However, a significant decrease (*p* < 0.05) in their migratory ability was observed following co-treatment with CDDO-me and FUS. In this treatment group (right panel), very little cell migration was seen at day 3. In Figure 3B, representative images of CFUs generated by C4-2B cells at two weeks post treatment are shown in the left panel. Bar graphs obtained from three independent experiments are shown in the right panel. As compared to controls, C4-2B cells exposed to either CDDO-me (100 nM) or FUS at level H4 were able to significantly (*p* < 0.05) suppress colony formation. Furthermore, combined treatment showed a significantly higher level of suppression in CFUs (*p* < 0.01). Indeed, very few colonies were documented in the combined treatment group. The effect of CDDO-me + FUS on spheroid formation by C4-2B cells was shown in Figure 3C. As evident from representative images of spheroids in the left panel, the untreated control cells formed nicely packed spheroids within two days. However, cells exposed to either CDDO-me (100 nM) or FUS (H4) showed loosely packed spheroid formation, and cells exposed to both treatments did not show any detectable spheroids. Data from multiple experiments are summarized in the bar graphs (right panel), which further corroborate that combined treatment fully suppressed the ability of C4-2B cells to form spheroids. 

The nuclear factor kappa-B (NF-κB) transcription factors play crucial roles in regulating the aggressive behavior of cancer cells and enable their survival post cytotoxic therapy [42]. Western immunoblot experiments were carried out to determine both basal and activated (phosphorylated) levels of NF-κB p65 in the control and treated C4-2B cells (Figure 3D,E). The GAPDH protein levels were used as internal controls. A representative image of the immunoblot is shown in the upper panel (top), and bar graph data on densitometric analysis, following normalization with GAPDH, are shown in the lower panel (bottom). Results indicated that the C4-2B cells that survived the cytotoxic effects of standalone FUS had higher levels of both basal and activated (phosphorylated) NF-κB p65. However, this increase in NF-κB levels was not seen in cells that were pre-exposed to CDDO-me (100 nM). This clearly indicated that CDDO-me + FUS suppresses the recurrence of aggressive phenotypes as compared to FUS alone. 

### 3.4. CDDO-Me Increases FUS-Mediated Suppression in Proliferation, Migration and Colonizing Ability and Enhances FUS-Induced Apoptosis and Necrosis in 22Rv1 Cells 

**Figure 4:**
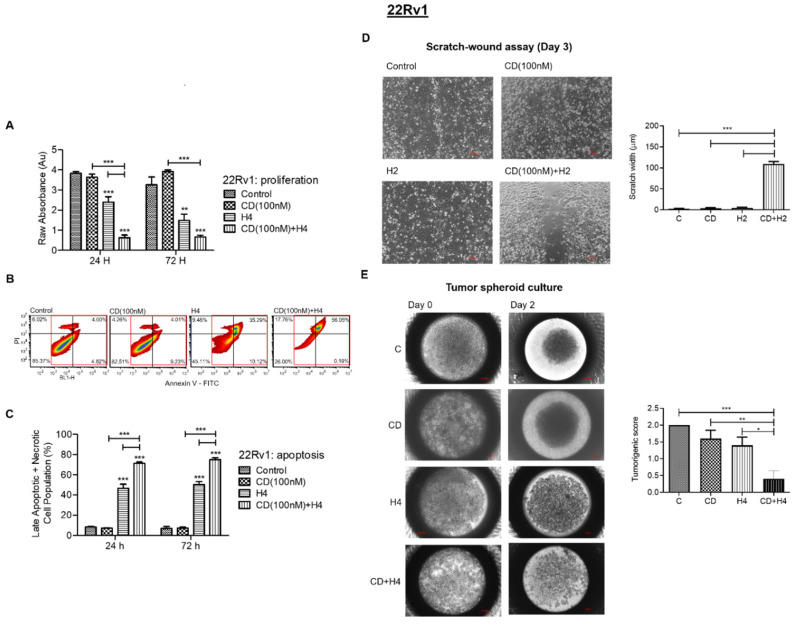
Effect of FUS, alone and in combination with CDDO-me, on cell viability, cell death, migration and spheroid formation in 22Rv1 cells. In (**A**), cell proliferation (WST-8 assay) at 24 h and 72 h post treatment with CDDO-me (100 nM) or FUS (H4), alone and in combination, is shown. (**B**) flow cytometry analysis of C4-2B cells exposed to CDDO-me (100 nM) or FUS (H4), alone and in combination. The percentage of early apoptotic/necrotic cells (PI+/FITC+) is shown in the upper right quadrant. The late apoptotic/necrotic 22Rv1 cells, as a percentage of total cell population, is shown in panel (**C**). (**D**) effect of low-dose FUS (H2), alone and in combination with CDDO-me (100 nM), at day 3 post treatment. Representative images of day 0 (top) and day 3 (bottom) wound width are shown on the left, and bar graph data on wound width at day 3 are on the right (*n* = 4). Data show a significant (*p* < 0.001) suppression in migratory properties of 22Rv1 cells following combined treatment. (**E**) effect of combination treatment (CD + H4) on spheroid formation by 22Rv1 cells at day 2 of the spheroid culture. Representative images of spheroids and bar graph data (*n* = 5) are shown on the left and right, respectively. Significant changes from untreated controls or individual treatment groups are represented as *p*-values (* *p* < 0.05; ** *p* < 0.01; and *** *p* < 0.001).

The 22Rv1 cell line is a model for aggressive CRPC cells that expresses the constitutively active AR variant, AR-V7 [43]. Progression to AR-V7-expressing PCa cells is known to dictate therapeutic resistance in clinical samples [44]. Therefore, we investigated the effect of standalone FUS (H4) and the enhancing effect of CDDO-me (100 nM) pre-treatment on cell viability, apoptosis and necrosis, migration and colonizing ability of 22Rv1 cells (Figure 4). Pre-treatment with CDDO-me (100 nM) significantly increased the FUS-mediated (H4) suppression in viability of 22Rv1 cells at both 24 h and 72 h (Figure 4A). Flow cytometry analysis also showed increased apoptosis/necrosis, as evident from 35.29% cell death with FUS alone as compared to 56.05% cell death following FUS and CDDO-me treatment (Figure 4B). The bar graphs in Figure 4C further corroborate the increases in late apoptotic and necrotic cells from ~40–45% with FUS alone to almost 80% in cells exposed to both FUS and CDDO-me (Figure 4C). In addition to the effect on 22Rv1 cell death, co-exposure to FUS and CDDO-me also suppressed the cell migration (Figure 4D) and spheroid-forming ability (Figure 4E) of the 22Rv1 cells. The bar graphs associated with each panel clearly show significant increases in FUS-mediated suppression of the aggressive properties in 22Rv1 cells pre-sensitized with CDDO-me.

### 3.5. In DU145 Cells, CDDO-Me and Nelfinavir Co-Exposure Further Increases FUS-Induced Oxidative Stress and Suppresses Their Aggressive Phenotype

**Figure 5:**
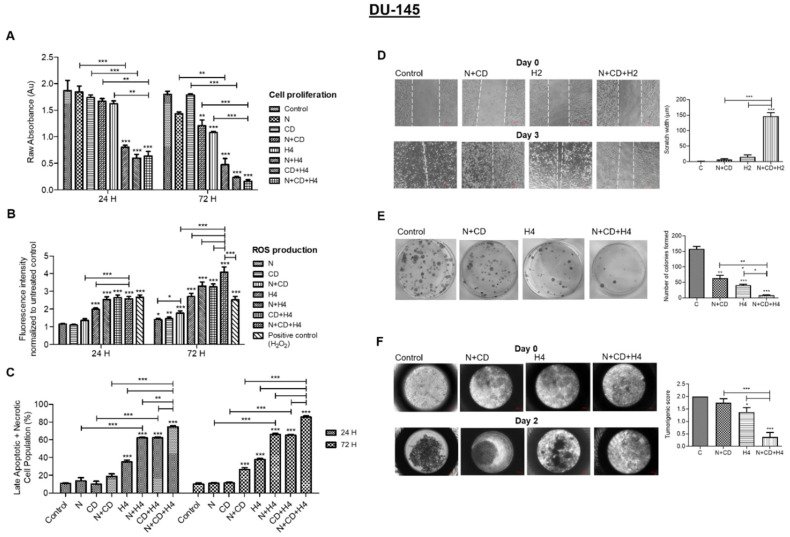
Effect of FUS, alone and in combination with CDDO-me and/or nelfinavir, on cell proliferation, cell death, ROS production, migration, colony formation and spheroid formation in DU145 cells. (**A**) DU145 cell proliferation at 24 h and 72 h post treatment with nelfinavir (N; 2.0 µM) and/or CDDO-me (CD; 100 nM), alone and in combination with FUS (H4). Pre-sensitization with both CDDO-me (Ox-stress) and nelfinavir (ER-stress) increases the anticancer effects of FUS. (**B**) ROS production by DU145 cells 24 h or 72 h post exposure to N, CD or N+CD, in the presence or absence of FUS (H4). As compared to individual treatments, the three-treatment combination significantly increases ROS production. DU145 cell death, as a percentage of total population, is shown in panel (**C**). Our three-treatment combination significantly increases the percentage of late apoptotic/necrotic cells. (**D**) effect of FUS (H2), alone and in combination with nelfinavir (2 µM) and CDDO-me (100 nM), on DU145 cell migration at day 3 post treatment. A representative image of day 0 (top) and day 3 (bottom) wound width is on the left, and bar graph data on wound width at day 3 are on the right (*n* = 4). (**E**) CFU assays in DU145 cells exposed to N+CD (2 µM and 100 nM) or FUS (H4). Representative images of colonies are on the left, and bar graph data (*n* = 3) are on the right. (**F**) effect of our three-treatment combination (N+CD+H4) on spheroid formation by DU145 cells at days post exposure is shown. Representative images of spheroids are on the left, and bar graph data (*n* = 8) are on the right. Significant changes from untreated controls or individual treatment groups are represented as *p*-values (* *p* < 0.05; ** *p* < 0.01; and *** *p* < 0.001).

The DU145 cells are a metastatic PCa line that do not express AR (AR-null) and possess a neuroendocrine phenotype [37]. In this highly aggressive cell line, we did not observe a significant increase in oxidative stress following exposure to increasing doses of CDDO-me alone (data not shown). Therefore, we first investigated whether ROS production by CDDO-me could be further augmented by co-exposure to nelfinavir, which is known to increase both oxidative stress and ER-stress. ROS fluorescence intensity measurements (Figure 5A) indicated that low-dose nelfinavir (N; 1.0 μM) or CDDO-me (CD; 100 nM) did not increase ROS as compared to controls (untreated); however, combined exposure (N + CD) clearly showed a 20–30% increase in ROS levels. Furthermore, as evident at 72 h post exposure, pre-sensitization with nelfinavir and CDDO-me (N + CD) significantly increased ROS production in DU145 cells following exposure to FUS (H4). As compared to cells exposed to two agents, N + H4 or CD + H4, a significantly (*p* < 0.01) higher ROS production was documented in cells exposed to all three treatments (N+ CD + H4). Next, we measured the effect of this combination treatment on the viability of DU145 cells at both 24 h and 72 h (Figure 5B). Compared to cells exposed to either drug alone, the cells treated with N+CD had a significantly lower viability, which was clearly evident at 72 h. Moreover, pre-treatment of cells with N+CD significantly increased the suppressive effect of FUS (H4) on cell viability. Interestingly, the increase in FUS-mediated cytotoxicity following sensitization with nelfinavir and CDDO-me was evident within 24 h, and this FUS-induced killing efficacy was further augmented at 72 h. Flow cytometry analysis further confirmed the significant increases in late apoptotic and necrotic cells (Figure 5C), as documented by 80–90% cytotoxicity, in the combined group at 72 h post treatment of the DU145 cells. 

In congruence with the effects of our combination regimen in LNCaP, C4-2B and 22Rv1 cells (Figure 1, Figure 2, Figure 3 and Figure 4), we observed a significant decrease in the aggressive behavior of DU145 cells as well (Figure 5D–F). Cell migration data at 48 h corroborated the aggressive properties of DU145 cells, as evident from a total wound closure in the untreated controls (Figure 5D). Although exposure to the drug combination (N + CD) or standalone FUS (H2) did not significantly suppress the migratory behavior of DU145 cells, we documented a significant suppression of migration in cells exposed to all three conditions (N + CD + H2). Similarly, data obtained with CFU assays also demonstrated the potent effect of our combination regimen (Figure 5E). At 14 days, the control group showed more than 150 colonies; however, the number of CFUs were decreased following exposure to the nelfinavir and CDDO-me combination (N + CD). Most interestingly, the number of CFUs generated by the DU145 cells was almost abrogated in the N + CD + H4 group. Similarly, the spheroid formation assay showed a profound effect of the combination regimen (Figure 5F). In control cultures, DU145 cells showed tightly packed spheroids at day 2, with an *in vitro* tumorigenic score of 2.0 (right panel). However, cells were loosely packed in the N+CD or H4 treatment groups, and no visible spheroid formation was observed in wells exposed to both drugs and FUS, and the tumorigenic score was <0.5. The above findings indicate that the combination regimen (N + CD + H4) has potent anticancer effect even in the highly aggressive DU145 cells. 

### 3.6. Pre-Treatment with CDDO-Me and Nelfinavir Decreases Both NF-κB and Akt Transcription Factor Levels in DU145 Cells 

**Figure 6:**
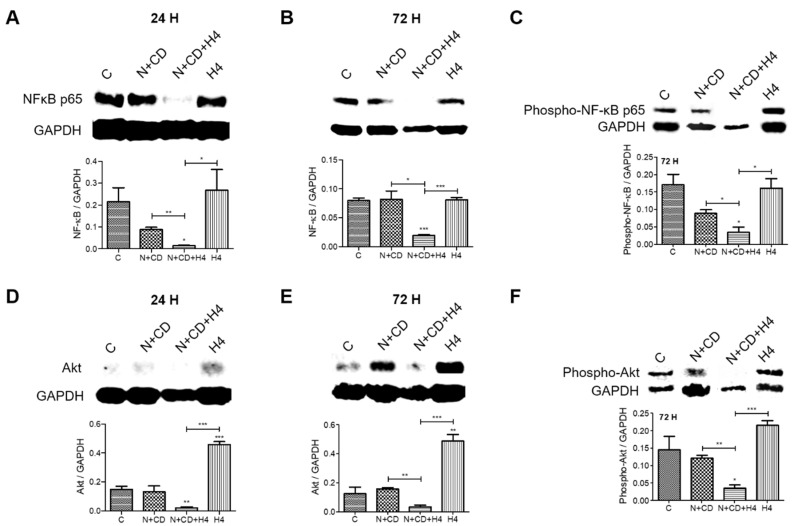
Effect of FUS, alone and in combination with CDDO-me and/or nelfinavir, on both total and activated NF-κB and Akt protein levels in DU145 cells. The effect of combined treatment with nelfinavir and CDDO-me (CD), alone or in combination with FUS (N + CD + H4), on both total and activated (phosphorylated) NF-κB p65 (**A**–**C**) and Akt (**D**–**F**) protein levels is shown. Representative immunoblot images are in the top panel, along with the loading control (GAPDH). Bar graphs in each of the bottom panels of A–F show normalized densitometric values. Data are representative of three independent experiments (*n* = 3). Significant changes from untreated controls or individual treatment groups are shown as *p*-values (* *p* < 0.05; ** *p* < 0.01; and *** *p* < 0.001). Unlike the recurrence of NF-κB and Akt levels in DU145 cells exposed to FUS alone (cf. Supplemental Figure S1), our three-treatment combination (N + CD + H4) abrogated the reactivation of these two crucial transcription factors.

To determine the molecular effects of our treatment combination on the aggressive behavior of DU145 cells, we carried out Western immunoblot experiments. The experiments determined both basal and activated (phosphorylated) levels of NF-κB p65 and Akt at both 24 h and 72 h post exposure (Figure 6). Similar to the data obtained in C4-2B cells (Figure 3), results obtained in DU145 cells also indicated that cells that survive standalone FUS exposure (H4) show increases in total p65 NF-κB level and its phosphorylation status (Figure 6A–C, cf. also Figure S1). However, this increase was not observed in cells that were pre-exposed to low-dose CDDO-me and nelfinavir. Although the N + CD treatment group showed a slight decrease in the total p65 phosphorylated NF-κB level, a more significant decrease was observed in the cells which survived the combined treatment regimen. The Akt transcription factor also plays a crucial role in the survival of cancer cells. Results obtained in DU145 cells showed that, similar to NF-κB, standalone FUS (H4) showed increases in both total Akt levels and Akt phosphorylation status in the surviving cells (Figure 6D–F). However, this increase was not documented in cells that were pre-exposed to low-dose CDDO-me and nelfinavir (N + CD), and both basal and activated NF-κB and Akt were barely detectable in the cells exposed to the combined regimen (N + CD + H4). Findings indicated that pre-exposure to CDDO-me (Ox-stress inducer) and nelfinavir (ER-stress inducer) suppressed the recurrence of aggressive phenotype and sensitized DU145 cells to lower FUS intensity. 

## 4. Discussion

The targeted, cancer-eliminating ability of FUS is being investigated in a number of different laboratories towards the development of more effective tumor-elimination approaches [45,46,47]. Ultrasound-guided, high-intensity FUS has shown promising results towards a safer approach in patients with tumors at difficult locations of the body [45]. However, the safety and efficacy of FUS needs to be thoroughly addressed before it can be regularly utilized in the clinical setting, especially against metastatic tumor foci. Orsi et al. (2020) presented published evidence on the efficacy of FUS on patients with metastatic hepatocellular carcinoma, colorectal cancer, breast cancer, neuroendocrine tumors, lymph node metastasis of breast cancer and metastatic pancreatic cancer [45]. By using a combinatorial strategy, using thermal ablation by FUS along with adjuvants and immune checkpoint blockade, Han et al. (2019) showed encouraging results on inhibitions in both metastatic tumor and tumor recurrence [46]. In a murine model of metastatic triple-negative breast cancer (TNBC), Sheybani et al. (2020) [47] showed that a tumor site targeted by a FUS regimen in combination with a systemically administered anticancer agent, gemcitabine, significantly decreased tumor outgrowth, improved survival and enhanced immunogenicity. Indeed, the tumor-eliminating ability of FUS was documented as early as 2004 by Abdollahi et al. [48]. Furthermore, in 2006, Uchida et al. demonstrated that FUS may be an effective and minimally invasive therapy for patients with localized PCa [38]. However, in majority of these past publications, the use of high acoustic intensities raised concerns regarding their long-term side effects. The threshold intensity below which FUS loses its destructive effect needs to be identified, and strategies to enhance the anticancer efficacy will be of great benefit in patients with highly aggressive, localized PCa [46,47,49]. 

In men with localized PCa, future considerations to augment the efficacy of localized FUS and its combination with systemic ADT may be warranted [5,6,50]. Both *in vitro* and *in vivo* models show that multiple signaling pathways are involved in the development of CRPC tumors [51]. Our *in vitro* findings using three different CRPC cell lines documented significant (*p* < 0.05) increases in the killing potential of FUS and suppression of aggressive phenotype. In C4-2B and 22Rv1 cells, we observed that the oxidative stress-inducing agent, CDDO-me, was sufficient in increasing the anticancer efficacy of FUS. However, in the highly aggressive, AR-null cell line, DU145, significant effects were only observed following sensitization with both CDDO-me and nelfinavir. In all four PCa cell lines, decreases in FUS-induced proliferation, migration and clonogenic ability and increases in FUS-induced apoptosis, necrosis and oxidative stress (ROS production) were observed following pre-sensitization. Therefore, our combined MCD regimen, i.e., pre-sensitization with stress-inducing chemical agents at clinically achievable levels, followed by exposure to targeted mechanical disruption via low-dose FUS (H4), may have profound translational potential. 

Treatment options for patients with CRPC are severely limited [5,37,39], and constitutively active AR splice variants, especially AR-v7, pose a significant challenge [52]. Our *in vitro* findings suggested that the above two orally available pharmaceutical agents may pre-sensitize CRPC cells and enhance the tumor-eliminating ability of FUS by suppressing crucial pro-survival signaling pathways. In addition to AR, both NF-κB and Akt signaling pathways play central roles in CRPC cell proliferation and metastasis, via pro-inflammatory signaling, and production of cytokines, chemokines and adhesion molecules [35,52,53]. In the absence of androgen, CRPC cells rely on these alternative pro-survival mechanisms to maintain their uncontrolled proliferation and metastatic behavior. We observed a similar induction in NF-κB and Akt signaling pathways in PCa cells that survived standalone FUS. Interestingly, however, this increase was not seen in the DU145 cells which were pre-sensitized with stress-inducing combination regimen, CDDO-me and nelfinavir. In addition, in 22Rv1 cells, we clearly demonstrated the therapeutic potential of our MCD approach against cells expressing the constitutively active AR-v7. Tumor cells are well known to cope with a variety of exogenous stresses via the activation of pro-survival mechanisms [54,55,56,57]. It is postulated that the threshold intensity needed for tumor elimination by FUS most likely increases with the aggressive properties of tumor cells, which enable the recurrence of tumors due to cancer cells that survive the initial FUS treatment. By disrupting both oxidative stress and ER-stress pathways, we were able to dysregulate the induction of survival mechanisms and enhance both apoptosis and necrosis following FUS exposure. 

**Figure 7:**
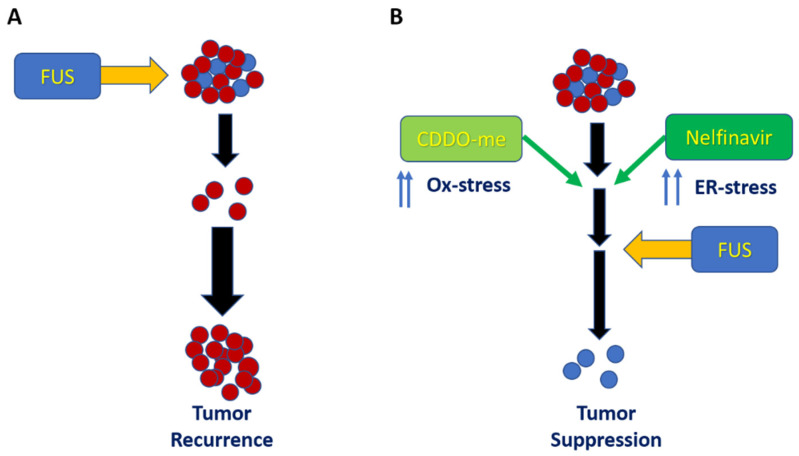
Putative effect of FUS alone (**A**) or pre-sensitization followed by FUS exposure (**B**) on tumor suppression. A combined mechanochemical disruption (MCD) approach may suppress the recurrence of aggressive cancer cells. Pre-sensitization of PCa cells with the oxidative stress (Ox-stress) inducer, CDDO-me, and the endoplasmic reticulum stress (ER-stress) inducer, nelfinavir, may increase the anticancer potential of low-dose FUS and decrease tumor recurrence.

Xiong et al. (2021) recently emphasized the crucial communications between these two stress-signaling pathways and their importance in cancer cells [54]. Farooqi et al. (2015) documented that multiple anticancer drugs can modulate both ER-stress and oxidative stress pathways to sensitize tumor cells [55]. Indeed, in two elegant publications, Cullinan et al. (2004 and 2006) showed that the coordination of ER-stress and oxidative stress signaling occurs via a novel signaling pathway, the PERK/Nrf2 axis [56,57]. Briefly, the antioxidant transcription factor, nuclear factor-erythroid factor 2-related factor 2 (Nrf2) is induced following oxidative stress and regulates protein synthesis via the ER. Stress in the ER induces the unfolded protein response (UPR) pathways via the activation of protein kinase RNA-like endoplasmic reticulum kinase (PERK), which dictates cell death [32]. Thus, Nrf2 signaling is required for survival during the UPR, and Nrf2 phosphorylation (activation) can be directly regulated via PERK. Recent studies have also shown that PERK signaling, via activation of both Nrf2 and activated transcription factor-4 (ATF4), can coordinate the convergence of ER-stress with oxidative stress signaling [56,57]. In this respect, the anticancer potential of targeting this PERK–Nrf2 signaling axis has been corroborated by a number of previous studies, both by us [32] and others [56], and suggests that, by combining CDDO-me, an oxidative stress and Nrf2 inducer [25,26], and nelfinavir, a potent ER-stress inducer [27,28], aggressive PCa cells can be sensitized to FUS therapy (Figure 7). 

Immunoblot studies showed that both basal and activated NF-κB and Akt levels are higher in the AR-null DU145 cells that survive standalone FUS, as compared to the AR-positive C4-2B cells (Supplementary Figure S1). This may help to explain why the significant cytotoxic effect of the two-treatment combination of CDDO-me and FUS, which was observed in the C4-2B cells (Figure 2 and Figure 3), was not evident in the DU145 cells. In DU145 cells, where both the NF-κB and Akt signaling pathways were activated following FUS, a significant reduction in cell viability and aggressiveness was attained by the triple combination regimen of CDDO-me, nelfinavir and FUS (Figure 5 and Figure 6). In this respect, studies have shown that the Nrf2-inducing effects of CDDO-me enable this drug to be a potent inhibitor of NF-κB [33]. Furthermore, in addition to its effects on ER-stress [26], nelfinavir is known to be a potent inhibitor of the PI3K-Akt signaling pathway [34]. The suppression of NF-kB at low-dose FUS and CDDO-me (H2 + CD) may be lower than that observed with the high doses of these agents (H4 + CD); however, since CDDO-me is known to be a potent inhibitor of NF-kB, we believe that the decrease in the migratory ability of CRPC cells may be due to the downregulation of NF-kB as well. Hence, there may be numerous advantages to using the above two NF-κB and Akt inhibitors to enhance the anticancer efficacy of FUS. Although exposure to CDDO-me acutely (30–120 min) increases ROS production to sensitize tumors to FUS in the long-term (12–24 h) by inducing Nrf2′s antioxidant effects, CDDO-me may be able to protect the normal cells from the oxidative stress generated by FUS. Future studies on the cytotoxic effects of our combined MCD approach on primary human cells would be needed to fully appreciate its translational significance *in vivo*. The second advantage of our combined MCD therapy would be to exploit the cross-talk between Ox-stress and ER-stress [33,34,56,57]. Our approach to pre-sensitize CRPC cells by targeting both of these stress-signaling pathways may reduce the ability of cancer cells to recur following FUS. Therefore, our future goals will be to further optimize the MCD approach towards a targeted elimination of metastatic PCa foci *in vivo*. In this respect, it should be emphasized that localized FUS delivery has already shown great promise for pain palliation in patients with bone metastases and has been approved by the FDA [58], and several clinically approved strategies using fluorescent reagents are available to clearly identify the metastatic tumor foci [59,60]. 

Although we have not monitored the effects of FUS, alone or in combination with CDDO-me and nelfinavir, in normal prostate cells, we do not believe that they would be toxic to surrounding normal tissue *in vivo* since we used subtoxic doses. Indeed, HIFU has been adapted for focal treatment of PCa with few adverse effects [61]. Our current studies used ultrasound at a much lower intensity as compared to the approved HIFU doses. With respect to the adverse effects of CDDO-me on normal epithelial cells, studies have shown that the antioxidant properties of low-dose CDDO-me may actually protect normal lung and breast epithelial cells from radiation [62]. Furthermore, our past publication using nelfinavir showed that the concentrations used in our experiments were not cytotoxic in either the prostate epithelial cell line, RWPE1 or in primary prostate epithelial cells (PrEC) [32]. 

## 5. Conclusions

We tested the *in vitro* effects of FUS, alone and in combination with CDDO-me or nelfinavir, in four PCa cell lines, i.e., LNCaP, C4-2B, 22Rv1 and DU145. As compared to standalone FUS, we documented significant suppressions in both survival and recurrence of PCa cells following this pre-sensitization. Thus, MCD therapy, in which localized FUS delivery is combined with systemically administered activators of stress-signaling pathways, may be a promising and translational strategy to ablate both localized and metastatic prostate tumors.

## Data Availability

The data presented in this study are available in article and supplementary material.

## Supplementary Materials

The following is available online at https://www.mdpi.com/article/10.3390/antiox11020341/s1, Figure S1: Standalone FUS at safe physiologic doses fails to downregulate the expression and activity of pro-survival markers NF-κB and Akt in CRPC cells.
