# Supplementary material for: Pre-Exposure to Stress-Inducing Agents Increase the Anticancer Efficacy of Focused Ultrasound against Aggressive Prostate Cancer Cells
Source: Antioxidants (Basel). 2022 Feb 9;11(2):341. doi: 10.3390/antiox11020341 (PMC8868501; doi:10.3390/antiox11020341)
Supplement: Supplementary file 1 [file antioxidants-11-00341-s001.zip › antioxidants-1511494-supplementary.pdf]

Supplementary Results

Standalone FUS Fails to Downregulate NF-κB and Akt in CRPC Cells

According to western blot analysis, the C4-2B cells constitutively expressed NF-κB but not Akt (untreated control group, Fig. S1AC), while the more aggressive DU-145 cells expressed both of these transcription factors (Fig. S1BC). Furthermore, the DU-145 cells had significantly higher ($p < 0.05$) expression of NF-κB (0.21 ± 0.06 per GAPDH) than the C4-2B cells (0.05 ± 0.01; Fig. S1C, left). Standalone FUS did not decrease the expression of NF-κB and Akt in the surviving C4-2B cells (0.96 ± 0.30 and 1.1 ± 0.93 of the untreated control level; Fig. S1D, left) and had no effect on NFκB expression in the surviving DU-145 cells (1.1 ± 0.28). A significant ($p < 0.001$) increase in Akt expression was observed in the DU-145 cells (3.4 ± 0.52).

The expression of phosphorylated (active) NF-κB was slightly stronger in the DU-145 cells than that in the C4-2B cells (0.17 ± 0.03 vs. 0.14 ± 0.05; Fig. S1C, right). Standalone FUS at level H4 did not diminish NF-κB signaling in the DU-145 cells (1.0 ± 0.31) and, in fact, it increased NF-κB activity in the C4-2B cells (2.5 ± 1.3; Fig. S1D, right) and enhanced the activity of Akt in the DU-145 cells (1.8 ± 0.65). These data indicate that FUS enables survival and rapid recurrence of PCa cells due to the increased expression and activity of NFκB and Akt pro-survival signaling pathways.

Supplementary Figures

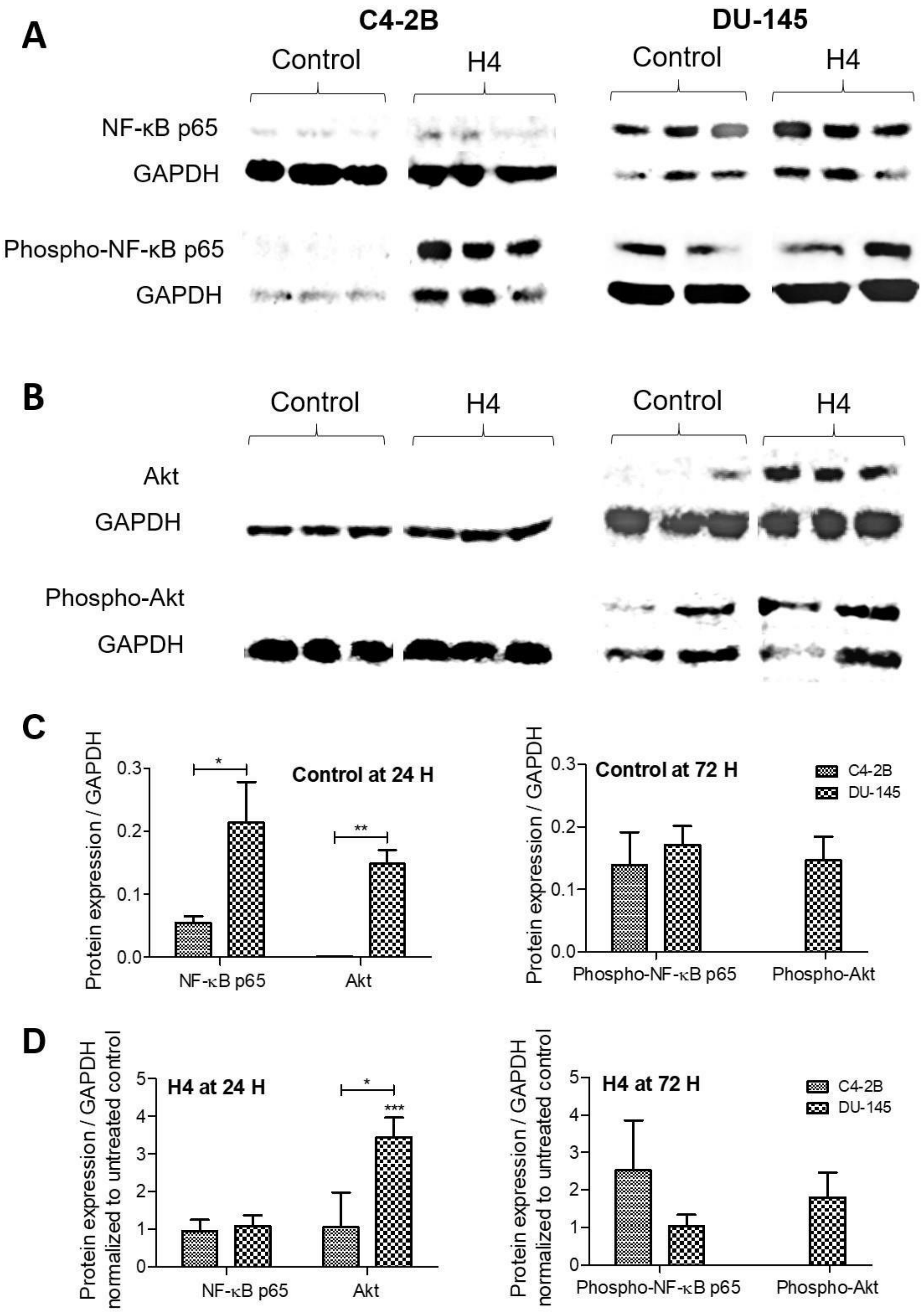

Figure S1. Standalone FUS at safe physiologic doses fails to downregulate the expression and activity of pro-survival markers NF-κB and Akt in CRPC cells. **A** and **B**, Shown are representative western blot images of the expression of total and phosphorylated NF-κB p65 (**A**) and Akt (**B**) in C4-2B and DU-145 cells, exposed or not to FUS at level H4. **C**, Expression of NF-kB p65 and Akt (left) and their phosphorylated forms (right) per GAPDH level for untreated C4-2B and DU-145 cells. **D**, Expression of NF-kB p65 and Akt (left) and their phosphorylated forms (right) per GAPDH and untreated control group levels for C4-2B and DU-145 cells treated by FUS at level H4. Total protein

expression data were collected at 24 h post-treatment, while phosphorylated protein expression was measured at 72 h. Values are mean ± SEM of 3-6 independent tests. *p<0.05. **p<0.01, ***p<0.001.
